# Bioenergy application of *Dunaliella salina* SA 134 grown at various salinity levels for lipid production

**DOI:** 10.1038/s41598-017-07540-x

**Published:** 2017-08-14

**Authors:** Rajper Aftab Ahmed, Meilin He, Rajper Asma Aftab, Shiyan Zheng, Mostafa Nagi, Ramadan Bakri, Changhai Wang

**Affiliations:** Jiangsu Provincial Key Laboratory of Marine Biology, College of Resources and Environmental Sciences, Nanjing Agricultural University, Nanjing, 210095 China

## Abstract

The biofuels are receiving considerable attention as a substitute for petro diesel. For microalgae, the cell density or biomass and lipid contents are key components for biodiesel production. This study was conducted to develop favorable culture conditions for *Dunaliella salina* to maximize its biomass and lipid accumulation. The effect of salinity (0.5 to 2.5 M NaCl) on the cell population, biochemical composition, and lipid output of *Dunaliella salina* was examined under a controlled environment for 21 days. Maximum growth (6.57 × 10^7^ to 7.17 × 10^7^cells mL^−1^) potentials were observed at 1.5 to 2 M NaCl. The photosynthetic pigments and carbohydrates also showed trends similar to growth. The maximum carotenoid level (5.16 mg L^−1^) was recorded at 2 M NaCl. Almost all physicochemical parameters increased with increases in salinity, biomass (1231.66 ± 1.26 mg L^−1^) and lipid content (248.33 mg L^−1^), as recorded at 2 M NaCl. Based on fluorescence intensity, the highest values (11.84 × 10^7^cells/ml) of neutral lipids and total lipids (22.28%) were recorded at optimum salinity levels. The present study suggests that a high biomass and lipid accumulation of *Dunaliella salina* SA 134 could be obtained at the 2 M NaCl level.

## Introduction

Energy is the fundamental requirement for the survival of mankind. Commercial development exists mutually with a growing world and has led to a large increase in the worldwide energy demand. If the governments all over the world adopt recent strategies, the world will require approximately 60% more energy in 2030 than it does today^1, 2^. At this time, approximately 90% of energy is produced from fossil fuels while only 10% is generated from new sources^3, 4^. The prominent concern with energy security led to the search for alternative sources that can decrease petroleum reliance to some extent^5^. Biofuels are such alternatives that have been emphasized by various countries as a fuel for the future. Biofuels have always been a key source of energy and have recently gained importance due to different environmental, geopolitical and economic reasons^6^. However, the conventional materials are still insufficient to produce enough fuel supply to fulfill the demands of the world^5^. Bio-energy possibly will contribute considerable to the world’s future energy supply, but its assembly requires land and water to do so^7^. Microalgae have been seen as encouraging pathway to produce bio-energy with limited (agricultural) land and water resources. High oil content and high photosynthetic conversion efficiency are other arguments in favor of microalgae^8, 9^. The maximum theoretical conversion efficiency of solar energy to microalgae biomass is 9%^10^, linked to a conversion competence of 2–3% of C4 plants. Microalgae are unicellular organisms, containing mainly of carbon, hydrogen, oxygen and nitrogen and are categorised as aquatic biomass. Day light availability is the supreme factor defining microalgae growth, followed by carbon and nutrients. Apart from bio-energy, microalgae can also be used for food, feed and chemical products^10, 11^.

Currently, microalgae are a potential source to produce viable third-generation biofuels^4^. Algae has gained keen interest since algae may possess more than 80% total lipids (instead of it rapeseed plants, which contain nearly 6% lipids). Different biofuel feed stocks and their oil production has been specified by different researchers Table 1.

**Table 1:** Some biofuel feed stocks and their oil production^12, 13^.

Crop	Oil yield (L/ha/year	Biodiesel Productivity (Kg/ha/year)	Crop	Oil yield (L/ha/year	Biodiesel Productivity (Kg/ha/year)
Soybean	446	562	Jatropha	1892	656
PalmOil	5950	4747	Sunflower	952	946
Rapeseed	1190	862	Microalgae^a^	58700	51927
Corn	172	152	Microalgae^b^	136900	121104

Algae ^a^comprises 30% oil (/wt) in biomass and Algae b 70%.

Microalgae can be the strongest substitute for fossil fuel in a cost-effective justifiable way and can also lead to decreased greenhouse gas production^14, 15^. The high photosynthetic ability of microalgae has not only useful for lipid growth but it is also useful as a capable practice for carbon sequestration, oxygen production and N cycling^16, 17^. Algae have the ability to alter their growth rate and their biochemical composition in various physiochemical states^18^. Many microalgae species are cultivated for the production of biofuel, industrial and pharmaceutical purposes at various stresses^19^. Lipids content in terms of percentage dry cell weight biomass of different micro algal species assumed by various scholars Table 2.

**Table 2:** Lipid content of different microalgae species^12, 13, 20–22^.

Microalgae species	Lipidcontent (% dry wt biomass)	Microalgae species	Lipid content (% dry wt biomass)
*Ankistrodesmus* sp.	24–31	*Monodus subterraneus*	16
*Botryococcus branuii*	25–75	*Monallanthus salina*	20–22
*Chaetoceros muelleri*	33	*Nannochloropsis oculata*.	22–29
*Chlamydomonas reinhardtii*	21	*Nannochloris* sp.	20–56
*Chlorella sorokiniana*	19–22	*Nannochloropsis* sp.	12–53
*Chlorella vulgaris*	5–58	*Neochloris Oleoabundans*	29–65
*Chlorella* sp.	10–48	*Pavlova salina*	30
*Chlorella protothecoides*	14–57	*Pavlova lutheri*	35
*Chlorella minutissima*	57	*Phaeodactylum tricornutum*	18–57
*Chlorella emersonii*	25–63	*Prostanthera incisa*	62
*Crypthecodinium cohnii*	20–51	*Prymnesium parvum*	22–39
*Dunaliella primolecta*	23	*Pyrrosia laevis*	69.1
*Dunaliella salina*	6–25	*Scenedesmus dimorphus*	16–40
*Dunaliella* sp.	17–67	*Scenedesmus obliquus*	11–55
*Dunaliella tertiolecta*	16–71	Schizochytrium sp.	50–77
*Ellipsoidion* sp.	27	*Skeletonema costatum*	13–51
*Euglena gracilis*	14–20	*Thalassiosira pseudonana*	20
*Haematococcus pluvialis*	25	*Isochrysis galbana*	7–40
Isochrysis sp.	7–33	*Nitzschia* sp.	45–47

Among these, *Dunaliella salina* is one such species that shows high lipid accumulation. *Dunaliella salina* has many capable characteristics that make it suitable for biodiesel production. Cells of the genus *Dunaliella* have no cell walls made of cellulose; furthermore, various laboratory techniques used to study intercellular mechanisms are more problematic to perform on cells with thick cell walls^23^. *Dunaliella* have certain benefits, for example, the disruption of cells is much easier than that in other algae because of the lack of a cell wall; they can also be easily cultured in laboratory conditions with a relatively high growth rate and an ability to be highly resistant to various environmental conditions compared to other algae. *D. salina* is present at various locations worldwide. They can tolerate areas with variable salinities ranging from 0.5 to 5 M NaCl by maintaining a gradually low intracellular ion concentration of ref. 24 and by forming compatible solutes such as glycerol, which maintains the structure and volume of the cell^25^. The ability to acclimate to changing salinities and its high metabolic and physiological changeability contributes to its proof of identity as a high potential for the high production of beta carotene^26^. Various studies have shown that growth^27^ and pigments^28^ of this alga are affected by salt stress conditions. It was noted that the beta-carotene to chlorophyll a ratio progressively increased with an increase in salt concentration, and as a result, the algae changed its color from green to deep orange^29^. *Dunaliella* sapecies are the best examples of microalgae that can tolerate high NaCl concentrations. Species of *Dunaliella*, such as *D. tertiolecta*, also produce large amounts of glycerol. Glycerol is made to increase cytoplasmic solute concentrations to increase the osmotic pressure of the cytoplasm and avoid fluid loss in saline solutions. Glycerol concentrations in the cell can be as high as 8 M^30^. Numerous species of *Dunaliella*, including *D. salina* and *D. tertiolecta*, produce large amounts of inner-thylakoid beta-carotene. Beta-carotene is a provitamin that is converted to vitamin A and is needed to form rhodopsin in the outer segment of rod cells in the eye. Beta-carotene also has antioxidant properties^31^. Because conditions that increase the lipid content of some species of *Dunaliella*, such as *D. salina*, also increase the beta-carotene content, marketing this product as a nutraceutical could offer another source of income for a biofuel company^32^. Many stress disorders have been measured with changed experimental formats to achieve ideal amounts of lipids for biofuels^33–35^. Salinity is the most important factor among all the environmental factors that affect the development, fatty acid content, and organic composition of microalgae^18, 36^. The salinity adaptability of the algae depends on the physiology of the species. Numerous physiochemical parameters affect the lipid formation of microalgae, such as intensity of light, pH, high or low temperature, nitrogen and salinity^37, 38^. The capability of *Dunaliella* species to bloom over the saturation range of salinities makes them a favorable candidate to study salinity effects on microalgae. The present study was an effort to detect the effect of various salt concentrations on the growth, biomass, lipid production and biopigment composition of *D. salina*. Cells of *D. salina* were transferred from 0 M to 2.5 M salinity in an artificial seawater medium^39^.

## Results

### Growth response of *D. salina* to various salinity levels

**Figure 1:**
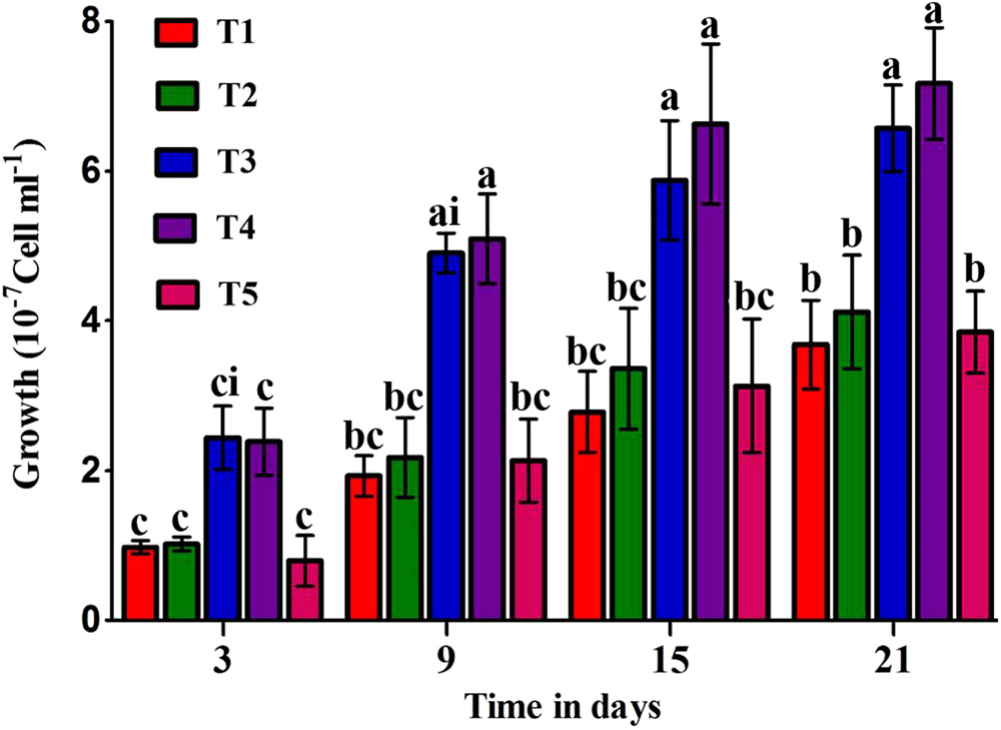
Cell density of *D. salina* cells grown over 21 d, with five treatments (T1 to T5) respectively at 0.5, 1, 1.5, 2.0, and 2.5 M NaCl. The analysis was done by two-way ANOVA considering Bonferroni post-tests to compare the means of the replicates at P < 0.05. Those labeled with different letters are significantly different.

As demonstrated in Fig. 1, growth of the cell population of *D. salina* grown at 0.5, 1, 1.5, 2 and 2.5 M NaCl for 21 d shows that the cell population increased in all treatments, but the highest values (6.57 × 10^7^ to 7.17 × 10^7^ cells mL^−1^) at the time of harvest were found at 1.5 to 2 M NaCl, the maximum salinity values for these microalgae. At T1 (0.5) and T2 (1) M NaCl, *D. salina* was exposed to a dilution pressure that meaningfully condensed its growth compared to the maximum level. In the meantime, T5 (2.5) M NaCl was an extreme salt stress that considerably restricted its biomass production. Meanwhile, there were no significant values found among T1, T2 and T5 during the whole experimental period; however, time had a significant effect on the treatments on days 3 and 21.

### Effect of salinity on the pigment content of *D. salina*

**Figure 2:**
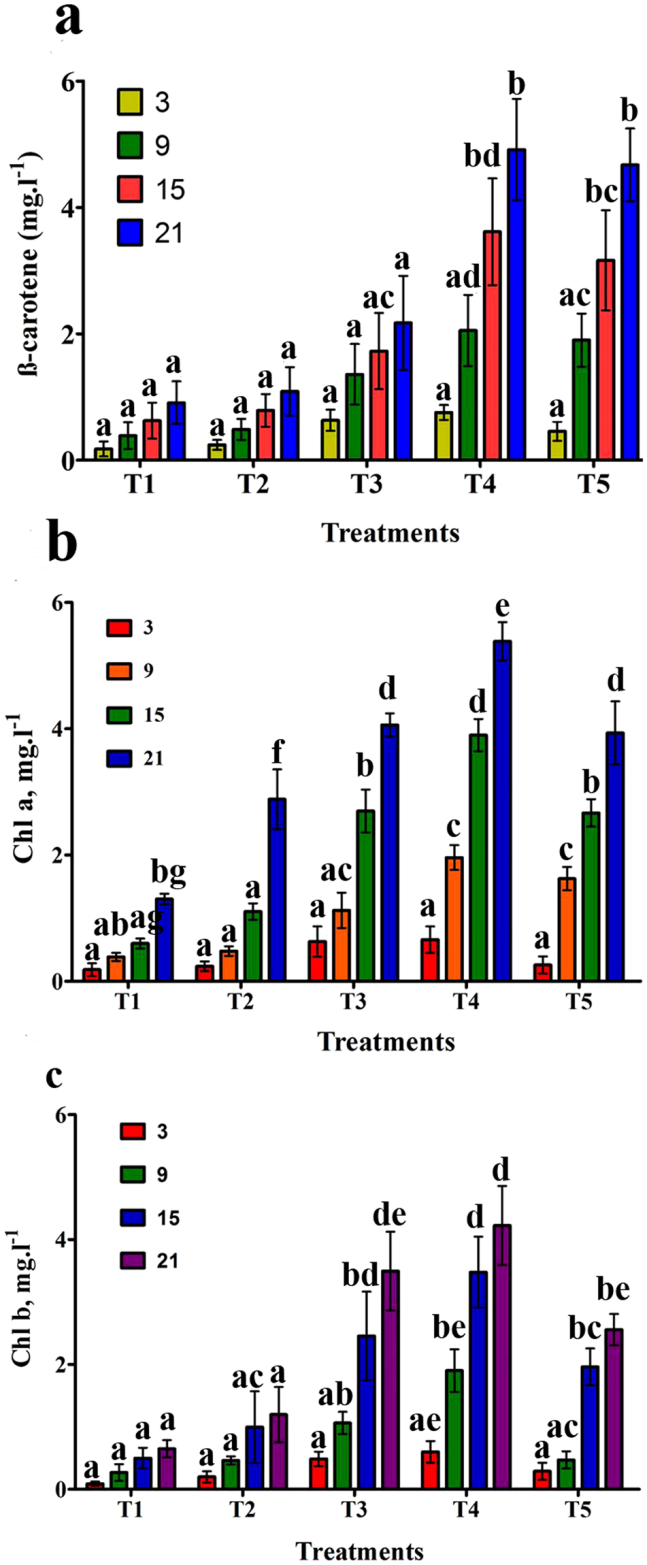
(**a**) Beta-carotene (mg L^−1^) in *D. salina* cells grown over 21 d, with five treatments (T1 to T5) respectively at 0.5, 1, 1.5, 2 and 2.5 M NaCl. (**b**) Chl a (mg L^−1^) in *D. salina* cells grown over 21 d at 0.5, 1, 1.5, 2 and 2.5 M NaCl. Points are the means of three replicates. (**c**) Chl b (mg L^−1^) in *D. salina* cells grown over 21 d at 0.5, 1, 1.5, 2 and 2.5 M NaCl. Points are the means of three replicates. Those labeled with different letters are significantly different according to Bonferroni post-test at P ≤ 0.05.

A variety of NaCl molarities (0.5 to 2.5) were used to study the pigmentation of *Dunaliella salina*. The content of all photosynthetic pigments (Beta carotene, Chlorophyll a and Chlorophyll b) were calculated every 6^th^ d of the experiment. The results illustrated in Fig. 2, a, b and c show that a significant change occurs in the beta carotene content among treatments T4 and T5 under different salinity levels compared to all others (T1, T2, & T3) and the highest values (4.916 and 4.673 mg L^−1^) were observed in treatments T4 and T5, respectively, on the 21^st^ d of the experiment.

#### Chlorophyll a

Figure 2b indicates that the highest value of chlorophyll a across all treatments was observed on day 21. Chl a accumulation had a minor but substantial increase from 1.302 mg L^−1^ to 5.380 mg L^−1^ with increasing salinity concentrations from 0.5 to 2.5 M representing the cell’s strategy for compensating for this stress by increasing its photosynthetic action.

#### Chlorophyll b

It was noted that with increasing time and NaCl concentration, an increase in Chl b production also occurred, and the significant difference between treatments has been shown in Fig. 2c. All mentioned photosynthetic pigments, stated in terms of mg L^−1^ culture, were highest at 2 M NaCl at the stationary phase of the experiment compared to T1 (0.5 M) and T5 (2.5 M) NaCl.

### Effects of NaCl shock on sugar construction

**Figure 3:**
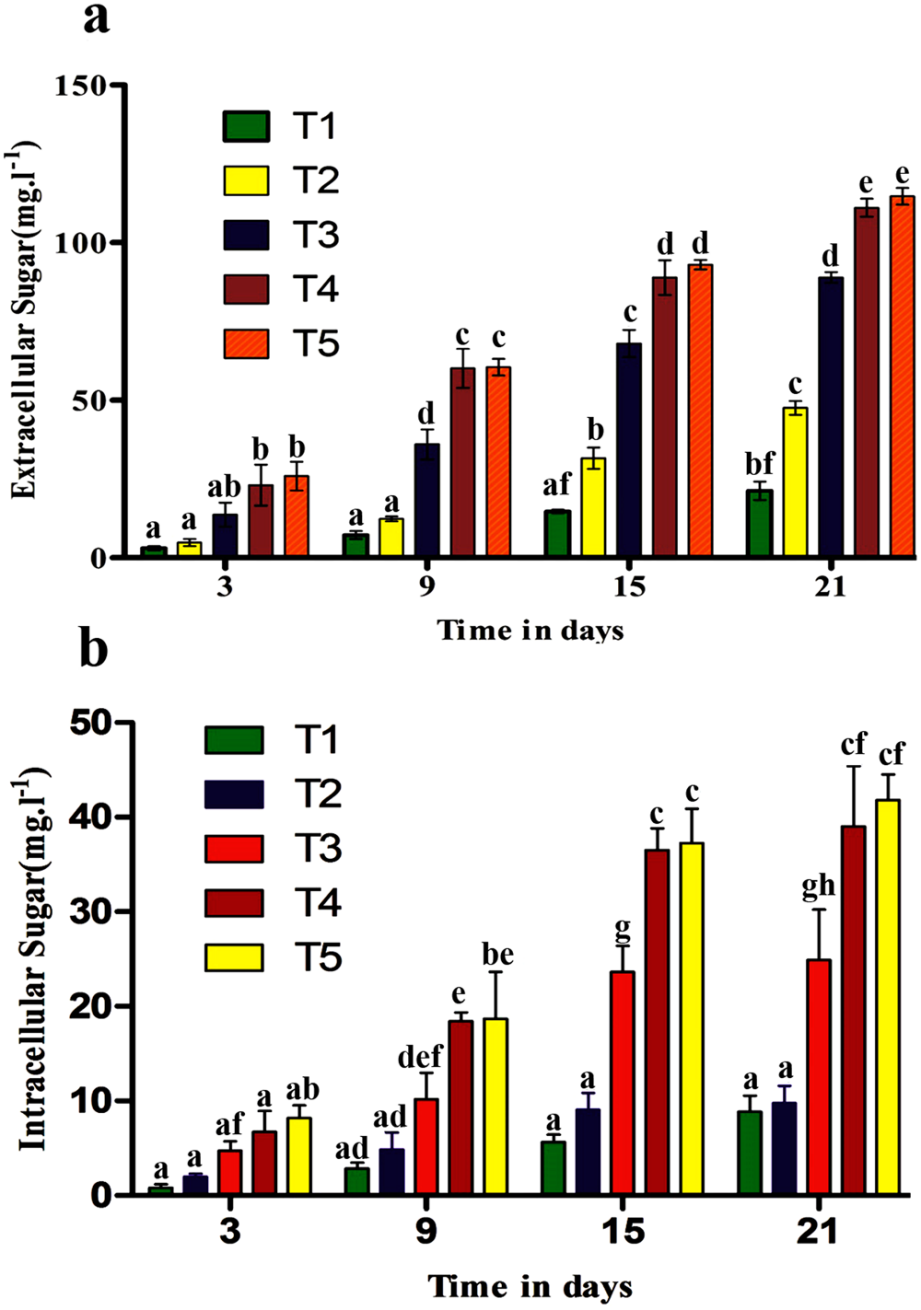
(**a**) Extracellular carbohydrate fraction (expressed as mg L^−1^) produced by *D. salina* grown over 21 d, with five treatments in the presence of different NaCl levels (0.5, 1. 1.5. 2 and 2.5 M) respectively. (**b**) Intracellular carbohydrate fraction (expressed as mg L^−1^) produced by *D. salina* grown over 21 d in the presence of different NaCl levels (0.5, 1. 1.5. 2 and 2.5). Points are the means of three replicates. Those labeled with different letters are significantly different according to Bonferroni post-test at P ≤ 0.05.

The data presented in mg L^−1^ culture in *D. salina* shows that total sugar (extra- and intracellular) production increased during the growth phase, whereas it was at its maximum at the stationary phase (Fig. 3a and b). This increase is similar in both the intracellular and extracellular sugars as both fractions presented a nearly similar trend. Analysis of both intracellular and extracellular sugars (Fig. 3a and b) revealed that the concentration of total carbohydrates matched the increased salinity levels. The highest extracellular as well as intracellular contents were recorded at the highest salinity (T5) level and the lowest content at the lower salinity levels (T1 and T2) on the 21^st^ day of the experiment (Fig. 3a and b).

### Changes in productivity over time

The cultures sampled at different ages between 3 and 21 days showed that as the age of the culture increases, the carbohydrate content progressively increased. The significant difference for both intra- and extracellular carbohydrates among all the treatments has been shown in Fig. 3a and b. The highest values were recorded at the stationary phase. There is, therefore, a strong interaction between *D. salina* carbohydrate productivity and time.

### Effect of salinity on neutral lipid production of *D. Salina*

**Figure 4:**
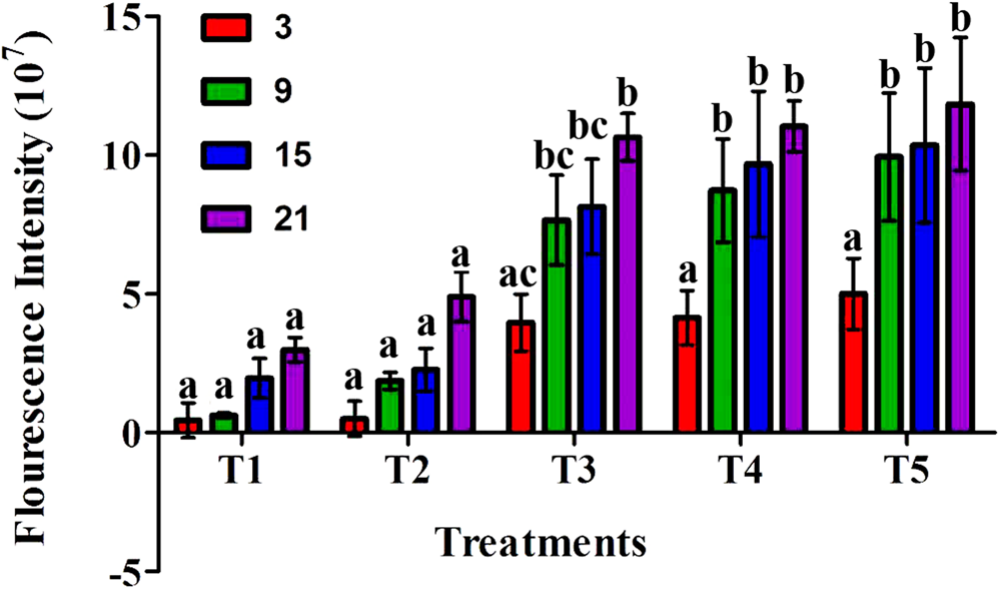
Nile red fluorescence intensity (expressed as 10^7^ cells ml^−1^) produced by *D. salina* grown over 21 d, with T1 to T5 respectively at different NaCl levels (0.5, 1. 1.5. 2 and 2.5 M). The analysis was done by two-way ANOVA considering Bonferroni post-tests to compare the means of the replicates; different labels at the bars show significant variation at P ≤ 0.05.

*D. salina* cells were grown for up to 21 d in media with different NaCl levels T1 (0.5 M) to T5 (2.5 M). Neutral lipid production was monitored every 6^th^ d using a microplate reader. We found that the increase in the salinity concentration, from 0.5 to 2.5 M, led to an increase in the total lipid content in the cells, in particular neutral lipids in the cells of *Dunaliella salina*. The highest fluorescence intensity, 11.835 × 10^7^ cells, was recorded at the highest salinity levels T5 at the end of the experiment, whereas lower intensity, 2.983 × 10^7^cells, was observed on the lowest salinity level T1 (Fig. 4). Time has a significant effect on T3, T4 and T5, as shown in Fig. 4 where there is no significant difference between T1 and T2 during the whole period of the experiment.

### Influence of salinity on *D. salina* biomass and lipid production

**Figure 5:**
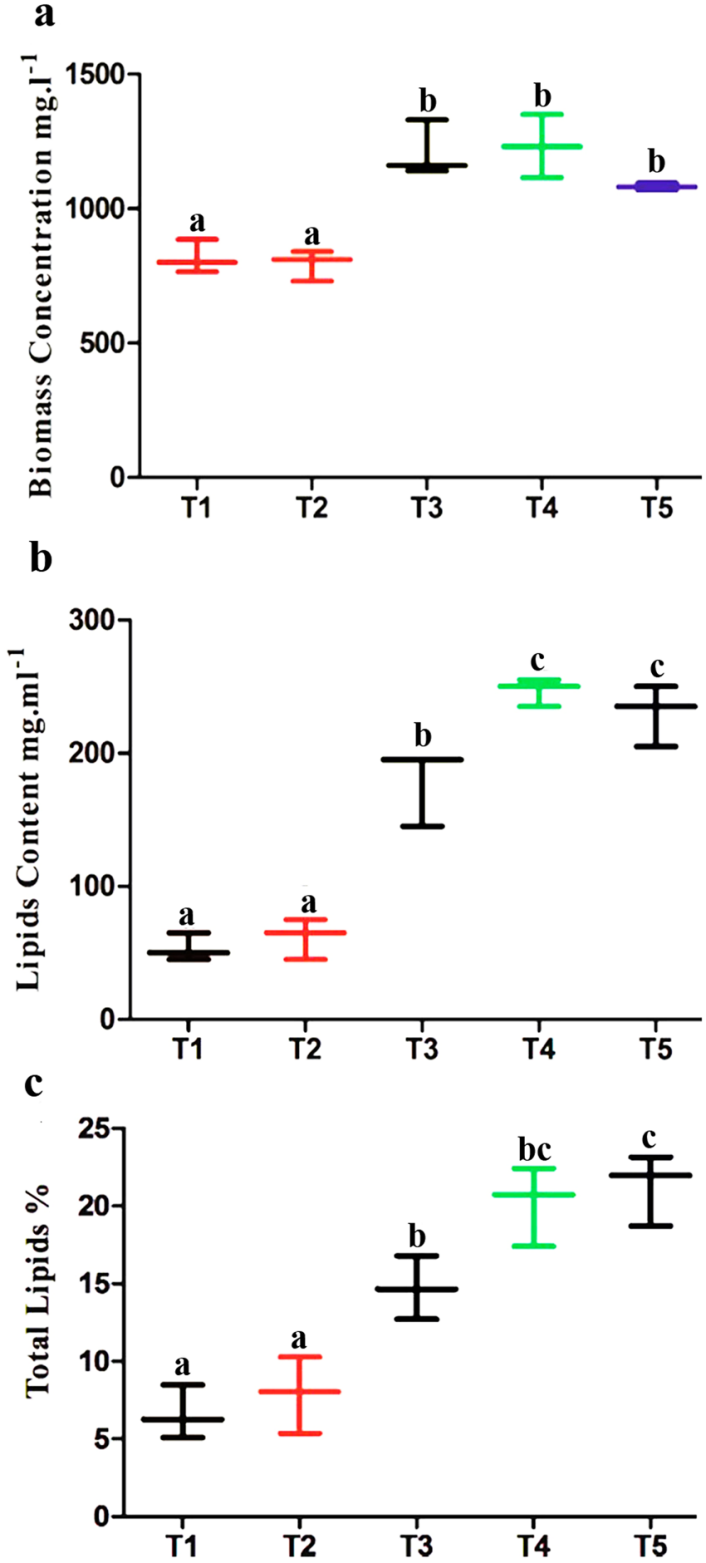
(**a**) Biomass of *D. salina* (mg L^−1^) grown over 21 d, with five treatments (T1 to T5) in the presence of different NaCl levels (0.5, 1. 1.5. 2 and 2.5 M) respectively. (**b**) Lipid contents of *D. salina* (mg L^−1^) grown over 21 d in the presence of different NaCl levels (0.5, 1. 1.5. 2 and 2.5 M). (**c)** Total lipid percentage of *D. salina* grown over 21 d in the presence of different NaCl levels (0.5, 1. 1.5. 2 and 2.5 M). Bars represent the means of three replicates. The analysis was done by two-way ANOVA considering Bonferroni post-tests to compare the means of the replicates; different labels at the bars show significant variation at P ≤ 0.05.

Normally, high salinity has a positive effect on microalga metabolite (such as fat production^40^). Likewise, salinity showed a significant progressive impact (p < 0.05) on biomass and lipid addition of *D. salina* (Fig. 5a–c). The result showed that the maximum biomass concentration (1231.66 ± 1.26 mg L^−1^, lipid content (248.33 mg L^−1^) was found at 2 M NaCl compared to all other treatments. The increase in lipid content at the maximum NaCl concentration may be due to an adaptation to stress conditions that helps in the accumulation of lipids; meanwhile, the total lipid percentage, 22.28%, was achieved at the highest salinity level, 2.5 M, compared to lower levels. It is a common trend that as biomass productivity decreases, the lipid per biomass percentage increases. This is evidence that salt stress significantly increases the percentage of lipids in *D. salina*, but it is also noted that as the overall biomass production of the culture decreases, the overall lipid productivity decreases.

## Discussion

Based on the cell amount and optical density at 680 nm, we perceived that *D. salina* showed its maximum growth at 1.5–2.0 M NaCl. At the lower concentration (0.5 M NaCl), cells were weakened by the stress, whereas at the higher concentration (2.5 M NaCl), they may face life-threatening conditions that may result in limited reproduction. These results are similar to those of ref. 41 who noted the valuable increase in growth of the species at 1.5 and 2 M NaCl and a reduction at lower salinity levels (0.6 M NaCl). For a shorter treatment time (16 d)^42, 43^, found the maximum growth of *D. salina* at 2 M NaCl and reduced growth at 3 M. Meanwhile, our results are in opposite to the ref. 44 who found the initial NaCl concentration higher than 1.0 M markedly inhibiting cell growth. However, these inconsistencies between their consequences and ours might be due to an intraspecific inconsistency. Comparable results were also reported by ref. 45 who found meaningful growth at 1.5 and 2 M NaCl^46^ and recommended that such changes in developmental forms in geographically distinctive species are due to the algae not adapting to a particular saline condition; however, *D. salina* can survive at a wider range of salinities. Other researchers^47^ stated that greater or lesser *Dunaliella* halophytic activity is inherent to each strain. Hence, lower growth proportions, at high salinity levels, cause larger cells to form and therefore more rapid nutrient reductions in the medium. Interactions between pigment content and NaCl have been studied by a number of researchers^28, 33, 48^ who stated that the quantities of beta-carotene and Chl a per ml of *D. salina* culture grown under constant illumination at 4500 and 11000 lux were at a maximum at 2.6 and 1.8 M NaCl, respectively. It has been formerly advised that beta carotene buildup through ROS defends cells contrary to the adverse effects of high salinity^49^. In the current study, Beta-carotene production by *D. salina* increased continuously with the increase in salinity, as shown in Fig. 2a. The maximum growth truly matched the highest chlorophyll and carotenoid concentrations, showing that the occurrence of salinity at a series of 2–2.5 M was required for enhanced pigment assembly. The present results support those of refs 41 and 45 who achieved greater chl and carotenoid concentrations at 1.5 and 2.0 M NaCl compared to lower NaCl levels. Moreover, chlorophyll differences with different NaCl concentrations showed a similar trend as total pigments; however, those carotenoids displayed a continuous increase up to 2.5 M.

Carbohydrate production is stimulated by stress environments^34, 50^. It was previously reported^34^ that soluble sugars have a vital role in the osmotic regulation of cells during reproduction and stress conditions. Among diverse solutes stored in response to stress, sugar may have important characteristics to sustain the osmotic regulation of cells. In the present study the salinity showed a positive effect on intracellular as well as extracellular sugars, meanwhile extracellular concentrations of sugars were greater than the intracellular. These results are similar to ref. 35, who stated that when the *Scenedesmus* sp. strain endured saline pressure, an increase in the extracellular sugar production from 22 to 650 mg/g p.s. was observed 24 h after the shock. Other researchers^36, 37^ also achieved high carbohydrate concentrations with a range of salinities mean while our results are more meaning full then^36^ stated on *Scenedesmus quadricauda*. It was evident from the results that neutral lipid production increased with the salinity levels. The maximum amount of the neutral lipids per cell was maximized at higher salt concentrations. The results are similar to ref. 51, who reported that by using NR fluorescence to identify neutral lipids, it became clear that when the salinity levels increased from 9% NaCl to higher NaCl levels, transitory neutral lipid presence is prompted; whereas those shifted to 15% NaCl growth medium stimulated the maximum neutral lipid addition, even though the cell division slowed down at maximum concentration. Therefore, the presence of neutral lipids might be subject to the preservation of cell division proficiency during the NaCl change from lower to higher levels. An inherent block in cell detachment that resulted in accumulation of neutral lipids in *Chlamydomonas* was previously presented^38^. Neutral lipids may have played a vital role in controlling the development of major structures in plants^52^. Numerous species of single-celled microalgae can store bulky volumes of TAG under nutrient and salt stress circumstances^53, 54^. The salt tolerant green alga *Dunaliella salina* (Teodoresco) is unique in that it stores, under high irradiances or nitrogen deficiency, huge quantities of plastidic lipid droplets, which are composed of TAG and two isomers of beta-carotene^55^. In conclusion, it is stated that by changing the salinity, the growth and neutral lipid formulations are significantly affected. The maximum lipid production can be achieved only by higher NaCl levels until cell development decelerates and cell separation is inhibited as a result of stress. The lipid production from green microalgae species generally possess a fatty acid profile containing predominantly C16 and C18 fatty acids, similar to that of vegetable oils and therefore fit for biodiesel production^56^. In general, greater salinity has a valuable effect on microalga metabolite (such as lipid) production^40, 54^. According to the results achieved in the present study, maximum cell growth, biomass concentration (1231.66 ± 1.26 mg L^−1^), and lipid content (248.33 mg L^−1^) were found at 2 M NaCl, while the lipid content per biomass percentage (22.28%) was observed at the 2.5 M NaCl levels. The present results are in accordance with^44^ who revealed that the addition of 0.5 and 1.0 M NaCl at the mid-log phase or the end of the log phase during cultivation significantly increased the lipid content, while, our results showed that increment of salinity up to 2.5 M significantly increased the lipid production. Presented results are more fruitful then^56^ found in *Nannochloropsis oculata and Chlorella vulgaris* for biodiesel production. Another study^57^ indicated that *Dunaliella* cells were reported to secrete glycerol in response to increases in NaCl concentration. The increase in lipid content might be associated with the adaptive response to high salinity concentrations, including cell volume changes and glycerol production.

## Materials and Methods

### Growth condition

Marine microalgae *Dunaliella salina* were purchased from Collection Centre of Marine Microalgae, Key Laboratory of Marine Ecology and Environmental Science (Nanhai Road, Qingdao 266071, China). The artificial medium was prepared for the growth and cultivation of microalgae and contained 0.42 g L^−1^ NaNO_3_, 0.0156 g L^−1^ NaH_2_PO_4_.2H_2_O, 0.044 g L^−1^ CaCl_2_.2H_2_O, 0.074 g L^−1^ KCl, 1.23 g L^−1^ MgSO_4_.7H_2_O, 0.84 g L^−1^ NaHCO_3_, and 0.0005 g L^−1^ Ferric Citrate. The trace element stock solution was prepared and each nutrient was added at following concentrations, 2.86 g L^−1^ H_3_BO_3_, 1.86 g L^−1^ MnCl_2_.4H_2_O, 0.22 g L^−1^ ZnSO_4_.7H_2_O, 0.39 g L^−1^ Na_2_MoO_4_.2H_2_O, 0.08 g L^−1^ CuSO_4_.5H_2_O and 0.05 g L^−1^ CO (NO_3_)_2_.6H_2_O. Fresh medium was prepared with different NaCl concentrations (0.5, 1, 1.5, 2, and 2.5 M), T1 to T5 respectively for experimental purposes. Cells were grown in flasks at 25 ± 1 °C under a 12:12 light-dark photoperiod with a light intensity of 42.4 µmol m^−2^ s^−1^ in artificial seawater as previously described^45^. Samples were shaken manually twice per day for gas exchange. Algae were grown in 500 ml Erlenmeyer flasks containing 300 ml liquid. Each experiment was conducted with three replicates.

### Measurement of the growth and pigments

Microalgae growth was observed by determining the optical density (OD) every 6^th^ day at a wavelength of 680 nm using a SpectraMax M5 microplate reader (Molecular Devices, US). The pigment content of every sample was determined as described by ref. 58. Two milliliters of the culture liquid was removed and centrifuged at 8000 rpm for 10 min. The pellets were extracted with 2 ml methanol at 4 °C for 24 h in the dark. The pigment contents were measured spectrometrically in methanol extracts at 470, 653 and 666 nm. Finally, the relative amounts of the pigments were calculated by the following equations:

1 $${\rm{Chlorophyll}}\,{\rm{a}}\,(\mathrm{Chl}\,{\rm{a}})={\rm{15}}{\rm{.65A666}}-{\rm{7}}{\rm{.34A653}}$$

2 $${\rm{Chlorophyll}}\,{\rm{b}}\,(\mathrm{Chl}\,{\rm{b}})={\rm{27}}{\rm{.05A653}}-{\rm{11}}{\rm{.21A666}}$$

3 $$\mathrm{Beta} \mbox{-} \mathrm{Carotene}\,({\rm{{\rm B}}}{\rm{c}})=({\rm{1000A470}}-{\rm{2}}{\rm{.86Ca}}-{\rm{129}}{\rm{.2Clb}})/\mathrm{245}$$

### Analysis of intra and extracellular sugars

A 5 ml sample was collected and centrifuged at 8000 rpm for 10 min. The supernatant was stored and used for extracellular sugar determination using the phenol, sulphuric acid method. Then, 5 ml of distilled water was added to the pellet and stored at −20 °C for 12 h, after which the sample was incubated at 37 °C for 20 min. This process was repeated 3 times. After that, the samples were centrifuged and 1 ml of supernatant was used for the intracellular sugar measurement with 0.5 ml phenol 6% plus 2.5 ml of concentrated H_2_SO_4_. The results were recorded spectrometrically at 490 nm.

### Analysis of neutral lipids

Nile red (9-dietylamino-5H-benzo (α) phenoxazine-5- one) staining was used to reveal the comparative amount of NL. The Nile red fluorescence strength of *Dunaliella salina* cells at different reproduction stages was analyzed by the improved method of ref. 59. Cell disruption (1 ml) was performed with 330 µL of 25% (v/v) Dimethyl sulfoxide (DMSO) watery solution and the mixture was processed by ultra sonication for 20 s. Then, 15 µL of 0.1 mg mL^−1^ Nile red was added to the mixture; after that, the cells were incubated at 40 °C for 10 min staining in a water bath. The radiation intensity of stained microalgae cells was noted with a Spectra Max M5 microplate reader (Molecular Devices, US) equipped with a 96-well plate reading method. Allowing the pre-scan of excitation and emission features of neutral lipid standards, the excitation and emission wavelengths of 475 nm and 570 nm were used. Each treatment was replicated three times.

### Effect of salinity on total lipid production

The dried biomass was extracted using 10 mL of methanol: chloroform mixture (2:1 v/v). The extract was centrifuged for 10 min at 8000 g to separate the cell-free organic phase. The procedure was repeated three times to maximize the purity of the lipids. Finally, the solvent phase was combined and evaporated to obtain the lipids. The lipid content was examined gravimetrically and stated as a dry weight %; the lipid production was considered.
